# Intercalation of Copper Phthalocyanine Within Bulk Graphite as a New Strategy Toward the Synthesis of CuO-Based CO Oxidation Catalysts

**DOI:** 10.3389/fchem.2020.00735

**Published:** 2020-08-31

**Authors:** Gaëlle Couvret, Ghislain Genay, Cerise Robert, Loïc Michel, Valérie Caps

**Affiliations:** ICPEES (Institut de Chimie et Procédés pour l'Energie, l'Environnement et la Santé), Université de Strasbourg - ECPM / CNRS UMR 7515, Strasbourg, France

**Keywords:** copper complexes, immobilization, controlled oxidation, catalytic CO oxidation, non-covalent interactions, exfoliation, graphite, graphene

## Abstract

Graphite is a widely available natural form of carbon with peculiar chemical and surface properties. It is essentially hydrophobic and consists in very stable stacks of graphene layers held together by highly delocalized π-π interactions. Its use in chemistry and in particular for catalytic applications requires modification of its structure to increase its surface area. This is commonly achieved by harsh oxidation methods which also modifies the chemical composition of graphite and enables subsequent deposition of catalytic phases via common impregnation/reduction methods. Here we show that copper phthalocyanine (CuPc) can be incorporated into unmodified bulk graphite by the straight-forward sonication of a dimethylformamide solution containing CuPc and graphite flakes. Immobilization of the CuPc complex in the graphitic matrix is shown to rely on π-π interactions between the Pc ligand and graphenic surfaces. This strong CuPc-graphene interaction facilitates oxidation of the graphitic matrix upon oxidation of the immobilized complex, as shown by thermogravimetric analysis in air. Nevertheless, a soft oxidation treatment can be designed to produce CuO nanoparticles (NPs) without degrading the dispersing graphitic matrix. These well-dispersed CuO NPs are shown (1) to decrease the degree of stacking of graphite in the solid-state by intercalation in-between graphitic stacks, (2) to be more easily reducible than bulk CuO, and (3) to be catalytically active for the oxidation of carbon monoxide. The higher mass-specific CO oxidation rates observed, as compared with CuO/alumina benchmarks, highlight the beneficial role of the carbon support and the relevance of this new strategy toward the design of copper oxide catalysts from copper phthalocyanine metal complexes.

## Introduction

Graphite is a naturally occurring, stable form of carbon. It is a highly crystalline, bulk, purely carbonaceous material which combines high conductivity, high thermal stability, exceptional mechanical resistance, resistance to corrosion, and lightness. It is thus widely used in its bulk form as electrodes and as component for refractories and for super-light materials. The world resource is estimated to be around 800 million tons (U.S. Geological Survey, Mineral commodity summaries 2020, p. 73), which by far exceeds resources in metals and other elements. On the nanoscale, it consists of stacks of graphene layers which are held together by strong π interactions due to electron delocalization over their extended aromatic networks (Wang et al., 2015). It exhibits virtually no surface area and its use as a catalyst support thus generally requires exfoliation and surface functionalization (Cai et al., 2012). Exfoliation allows to break the stacks down to thinner stacks, ultimately to single layer graphene if needed, hence increasing the available surface which can be used for subsequent deposition of metal nanostructures. Surface functionalization aims at providing reactive sites for decoration with such active phases.

The most widely used exfoliation/functionalization method consists in harsh oxidation of the graphitic structure by strong acids, sodium nitrate, and potassium permanganate following Hummers-type procedures (Hummers and Offeman, 1958). In this approach, deep exfoliation of the structure results from a drastic change in the chemical composition of the planes, namely the introduction of out-of-plane, covalently-bound oxygen atoms (Dreyer et al., 2014). The increased interlayer spacing is retained in the dry state. The resulting graphite oxide is water-soluble and can further react with metal salts in solution. Metal ions can thus easily be anchored onto the structure via surface oxygen groups. Graphene-supported metal nanoparticles are subsequently obtained in a third step by co-reduction of the graphitic oxide matrix and the interacting metal ion (Goncalves et al., 2009).

Organic dyes are also known to exfoliate and functionalize graphite by interacting non-covalently with graphene layers (Schlierf et al., 2013). They stabilize graphene suspensions by intercalation between the sonically-destabilized basal planes, generating 3D systems in solution and hybrid systems in the solid-state. However, it is interesting that, when it comes to metal-containing dyes, such as copper phthalocyanine (CuPc) for example, most work related to the chemical synthesis of functional CuPc/carbon composites involve graphite oxide (Chunder et al., 2010; Mensing et al., 2012; Mukherjee et al., 2017) and high surface area, functionalized carbons (Ding et al., 2012; Goenaga et al., 2014; Reis et al., 2014). There is to our knowledge no example of direct chemical deposition of CuPc on low surface area, bare hydrophobic graphite via non-covalent interactions. Besides, the reported carbon-supported CuPc are either used as such, i.e., with no further transformation of the immobilized CuPc complex (Pakapongpan et al., 2014; Reis et al., 2014; Bian et al., 2020), or after pyrolysis at high temperatures to modify the carbon support (Ding et al., 2012; Goenaga et al., 2014). Their main applications are electrochemistry (Ding et al., 2012; Goenaga et al., 2014; Pakapongpan et al., 2014; Reis et al., 2014; Bian et al., 2020) and photocatalysis (Mukherjee et al., 2017). When associated with other active supports, CuPc are also used for catalytic oxidations (Raja and Ratnasamy, 1996; Sorokin, 2013) and as co-catalyst for photoelectrochemical water splitting (Li et al., 2019). However, there is, to our knowledge, no example of the use of CuPc as a precursor to a catalytically active Cu or CuO phase (Gawande et al., 2016).

Due to their redox chemistry, copper oxides are known to catalyze useful reactions such as the oxidation of CO (Wan et al., 2008; Gawande et al., 2016). This reaction is of both practical importance (Waikar et al., 2019) and academic interest (Caps et al., 2008; Freund et al., 2011). It is indeed used for air depollution, as CO is toxic to human health (Soliman, 2019), and for purification of hydrogen fuel cells gas feed (Ivanova et al., 2010; Laveille et al., 2016). It is also a quite simple redox reaction that is catalyzed by metal oxides following a Mars van Krevelen mechanism (Mars and van Krevelen, 1954). It can thus also be used to probe the reducibility of oxide catalysts (Laveille et al., 2013).

Here we show that an efficient CuO/graphite CO oxidation catalyst can be prepared by soft oxidation of a strongly interacting CuPc/graphite precursor, with no need for extensive exfoliation of graphite. First, we investigate the potentials of metal complexes, namely copper phthalocyanine and copper tetraphenylporphine (CuTPP), as precursors for the direct decoration of unmodified hydrophobic bulk graphite with functional copper phases, by screening various sonication-based, organic exfoliation conditions in the liquid phase. Then, by combining thermogravimetric analysis (TGA) and Fourier-Transform Infra-Red (FTIR) studies, we show that copper phthalocyanine strongly interacts with the graphitic surface and that it can be thermally oxidized to an easily reducible, catalytically active cupric oxide phase, which is active for CO oxidation both in the absence and in the presence of hydrogen.

## Materials and Methods

### Chemicals

Copper (II) phthalocyanine (CuPc, >95%, Alfa Aesar, MW 576.07 g/mol, 11 wt.% Cu), 5,10,15,20-tetraphenyl-21H,23H-porphine copper (II) (CuTPP, Sigma Aldrich, MW 676.27 g/mol, 9.4 wt.% Cu), copper nitrate [CuNit, Cu^II^(NO_3_)_2_.xH_2_O, Puratronic, 99.999% metals basis, Alfa Aesar, MW 187.55 g/mol (anhydrous)], N,N-dimethylformamide (DMF, >99.8%, Alfa Aesar), 1-methyl-2-pyrrolidone (NMP, ≥99.8%, Alfa Aesar), sodium borohydride (NaBH_4_, ≥98%, Sigma Aldrich), graphite powder (NG, natural, high purity 200 mesh, 99.9999% metal basis, ultra-superior purity, Alfa Aesar) were used without further purification.

### Synthesis Using High Power Sonication

High purity graphite (200 ± 2 mg) was dispersed in 200 mL of DMF and sonicated using a Bandelin HD2200 probe sonicator (200 W, 20 kHz), at 50 or 20% of its maximum power, during a time t_0_ = 5 min. The Cu precursor was then added (181 mg for CuPc, 213 mg for CuTPP and 59 mg for copper nitrate, 20 mg/0.315 mmol/1.6 × 10^−3^ M Cu, Cu/NG = 0.10 w./w., i.e., Cu/(Cu+NG) = 9.1% w./w.), after which the mixture was further sonicated for a time t_1_ = 5 or 35 min. The solid was recovered by hot filtration (final synthesis temperature ~46–67°C) and dried in air for 16–24 h at 100°C. For copper nitrate, one synthesis was performed in the presence of sodium borohydride, as previously described (Michel et al., 2019). In this case, a given amount of NaBH_4_ (119 mg/3.15 mmol/1.6 × 10^−2^ M, NaBH_4_/Cu = 10) was added before recovery of the solid and the mixture was further sonicated for a time t_2_ = 5 min. Final temperature was 82°C. The solid was then recovered by hot filtration and dried in air for 24 h at 100°C. After cooling down to 22°C, the sample was washed with deionised water (2^*^200 mL) and dried in air for 24 h at 100°C.

One CuPc/graphite composite was subsequently calcined in 10% O_2_/He under the following conditions: 100 mL/min, 5°C/min, 400°C, 30 min. Final composites are denoted *X%Cu precursor*/graphite[*Y*], where *X%* stands for the copper weight content, *Cu precursor* stands for the copper precursor used (CuPc, CuTPP, or Cu(NO_3_)_2_) and *Y* stands for the main synthesis parameters, i.e., sonication power, contact time, use of NaBH_4_, final temperature in Celsius of the heat treatment. Six composites were synthesized: CuPc/graphite[50%/5 min], CuPc/graphite[50%/5 min/400], CuPc/graphite[20%/35 min], CuTPP/graphite[20%/35 min], CuNit/graphite[20%/35 min], CuNit/graphite[50%/5 min/NaBH_4_].

### Synthesis Using Low Power Sonication

This procedure was previously described (Vigneron et al., 2014). High purity graphite (100 ± 1 mg) was dispersed in 55 mL of DMF and sonicated using a Bandelin HD2200 probe sonicator (200 W, 20 kHz), at 20% of its maximum power, during a time t_0_ = 5 min. The beaker containing the mixture was then transferred to a sonication bath (Elma, ElmasonicOne, 30 W, 35 kHz). The bath contained 200 mL of water. The beaker was placed and maintained in a specific position within this water bath to ensure repeatability of the preparation (Vigneron et al., 2014). The mixture was allowed to cool down to room temperature (about 22°C) before the Cu precursor was added (92 mg for CuPc and 107 mg for CuTPP, 10 mg/0.16 mmol/0.8 × 10^−3^ M Cu, Cu/NG = 0.10 w./w., i.e., Cu/(Cu+NG) = 9.1% w./w., unless otherwise mentioned). The sonication bath was then turned on and the mixture allowed to sonicate for a time t_1_ = 60, 120, or 240 min. The solid was recovered by hot filtration (final synthesis temperature ~53–71°C) using nylon membrane filters (0.45 μm pore size) and dried in air for 21 h at 100°C.

Two *Cu precursor*/graphite composites were subsequently calcined in static air using a muffle furnace under the following conditions: 10°C/min, 450°C, 2 h. Final composites are denoted *X%Cu precursor*/graphite[*Y*], where *X%* stands for the copper weight content, *Cu precursor* stands for the copper precursor used (CuPc or CuTPP) and *Y* stands for the main synthesis parameters, i.e., type of sonication (bath), contact time, initial Cu/NG ratio when it is decreased to 1 wt.%, solvent when it is changed to NMP, final temperature in Celsius of the heat treatment. Eleven composites were synthesized, as detailed in Table 1.

**Table 1:** Main sonication parameters used for the synthesis of graphite-immobilized copper complexes, resulting Cu content and thermal stability of the synthesized composites.

**Entry**	**Composite**	**Sonication conditions**		**Cu content (wt.%)^a^**	**Targeted Cu content (wt.%)**	**Deposition efficiency (%)^b^**	**T_1/2_ (°C)^c^**
		**Power (W)**	**Time**				
1	CuPc/graphite[50%/5 min]	100	5′-5′	3.5	5.2	67	n.a.
2	CuPc/graphite[20%/35 min]	40	5′-35′	4.6	5.2	88	647
3	CuPc/graphite[bath/60 min]	30 (bath)	5′-60′	6.4	5.2	100^f^	633
4	CuPc/graphite[bath/120 min]	30 (bath)	5′-120′	5.5	5.2	100^f^	658
5	CuPc/graphite[bath/60 min/1%]^d^	30 (bath)	5′-60′	0.69	0.94	73	742
6	CuPc/graphite[bath/120 min/1%]^d^	30 (bath)	5′-120′	0.76	0.94	81	742
7	CuTPP/graphite[20%/35 min]	40	5′-35′	5.2	4.9	100	668
8	CuTPP/graphite[bath/120 min]	30 (bath)	5′-120′	3.9	4.9	80	730
9	CuTPP/graphite[bath/240 min]	30 (bath)	5′-240′	3.0	4.9	61	735
10	CuTPP/graphite[bath/240 min/NMP]^e^	30 (bath)	5′-240′	0.62	4.9	13	757
11	CuTPP/graphite[bath/120 min/1%]^c^	30 (bath)	5′-120′	0.16	0.89	18	774
12	CuTPP/graphite[bath/240 min/1%/NMP]^d, e^	30 (bath)	5′-240′	0.06	0.89	7	785
13	CuNit/graphite[20%/35 min]	40	5′-35′	0.02	9.1	0.2	753
14	CuNit/graphite[50%/5 min/NaBH_4_]	100	5′-5′	0.7	9.1	8	n.a.
15	CuPc/graphite[50%/5 min/400]	100	5′-5′	7.8	-	-	n.a.
16	CuPc/graphite[bath/60 min/1%/450]^d^	30 (bath)	5′-60′	0.77	-	-	n.a.
17	CuTPP/graphite[bath/240 min/1%/NMP/450]^d, e^	30 (bath)	5′-240′	0.06	-	-	n.a.

a *Based on ICP measurements (a relative standard deviation of ±5% applies to the reported values), except for entries 15–17. For the last 3 entries, the Cu content is estimated by attributing the weight loss measured after calcination to carbonaceous material (Michel et al., 2019)*.

b *Deposition efficiency is the ratio, between the real Cu content as measured by ICP and the targeted Cu content based on the Cuprec/(Cuprec+graphite) weight ratio initially present in the synthesis mixture, i.e., 5.2, 4.9, and 9.1 wt.% for CuPc, CuTPP, and CuNit-based composites, respectively*.

c *TGA-derived temperature of half decomposition of graphite defined as the maximum of the weight loss vs. temperature derivative above 500°C (derivatives of the curves presented in Figure 1, not shown). It corresponds to the temperature at which 50% of graphite is oxidized*.

d *Synthesis using an initial Cu/graphite ratio of 1 wt.%*.

e *Use of NMP instead of DMF as synthesis solvent*.

f *The excess copper found by ICP is attributed to loss of unreacted graphite on the walls of the beaker upon slight evaporation of the DMF solvent at the extended reaction times*.

### Elemental Analysis (ICP)

Elemental analysis (ICP) of the composites was performed by Inductively Coupled Plasma Optical Emission Spectroscopy (ICP-OES, Varian 720ES) at IPHC/CNRS UMR7178 (France). Deposition yields (deposition efficiencies) are defined as the ratio between the copper content determined by ICP and the initial Cu/(Cu+NG) value introduced in the synthesis mixture.

### X-ray Diffraction (XRD)

X-ray Diffraction (XRD) patterns of CuO@NG composites were collected on a Bruker D8 Advance theta-theta diffractometer (Cu Kα radiation, λ = 0.154 nm), equipped with a LynxEye detector and operating at 40 kV and 40 mA. The datasets were acquired in step-scan mode over the 25–58° 2θ range, using a step interval of 0.0132° and a counting time of 1 s per step. Samples were prepared by weighing out 50 mg of catalyst and pressing at about 6,300 kg/cm^2^ (Eurolabo press, 5 t), to get flat and smooth pellets which were then deposited on round-shaped glass plates. Average size of CuO crystallites (d_CuO_) was determined by the Scherrer equation, using the full widths at half-maximum (β = π × FWHM/180) of the CuO (002) and (111) reflections at 2θ = 35.5 and 38.7°, respectively:

$$\begin{array}{l} d_{\text{CuO}}=\left(\frac{0.9\;\lambda }{\left(\beta_{\text{CuO}\left(002\right)}cos\theta_{\text{CuO}\left(002\right)}\right)}+\frac{0.9\lambda \;}{\left(\beta_{\text{CuO}\left(111\right)}\text{cos}\theta_{\text{CuO}\left(111\right)}\;\right)}\right)/2 \end{array}$$

Slight differences in peak positions were observed [e.g., 26.3 ± 0.4° for C(002)]. They were attributed to slight differences in the z and corrected by adjusting the position of the C(002) peak maximum at 26.5°.

### Fourier Transform Infrared (FTIR)

Fourier transform infrared (FTIR) spectra were recorded by Attenuated Total Reflectance with a Thermo Fisher Nicolet iS10 spectrometer in the 525–4,000 cm^−1^ range. Powders (few mg) are placed directly on the 2 × 2 mm diamond window and cover the whole surface.

### Thermogravimetric Analysis (TGA)

Thermogravimetric analysis (TGA) was performed on a TGA Q5000 from TA Instruments. The powder sample (1.0 ± 0.1 mg) was placed in a Pt pan, which was then heated to 1,000°C at 10°C/min under flowing air (25 mL/min). Evolution of weight loss was monitored by the TA Qseries software.

### Temperature Programmed Reduction (TPR)

Temperature programmed reduction (TPR) was carried out on an AutochemII Chemisorption apparatus from Micromeritics equipped with a thermal conductivity detector. The sample (23 mg) was loaded in a quartz tube and heated to 900°C under a flowing mixture of 10% H_2_/Ar (5 mL/min) using a temperature ramp of 5°C/min.

### Scanning Electron Microscopy (SEM)

Scanning Electron Microscopy (SEM) was carried out on a Zeiss Gemini SEM 500 system at 10 kV using a working distance (WD) of 13.3 mm and an aperture (AP) of 30 μm, unless otherwise mentioned. Two detectors were used alternatively: an in lense secondary electron detector (In Lens) for best imaging resolution and a Everhart-Thornley detector (SE) for the detection of secondary electrons providing both topography and chemical contrast, with the lightest elements (C) appearing darker than the heaviest elements (Cu). An EDXS SDD probe (30 nm^2^) confirmed that C, Cu, and O were the only elements present in the composites and that the white spots were indeed copper oxides. The powder samples were pressed on a sample holder decorated with carbon tape before analysis.

### Catalytic Evaluation

Catalytic evaluation was performed in a fully automated (CETRIB SARL, Andlau, France) fixed-bed flow reactor (i.d. 10 mm) loaded with 26 mg of composite (0.26–2.5 mg CuO/3.2–32 μmol Cu). The gas mixtures balanced in He, i.e., 1% CO/1% O_2_ for CO oxidation and 1% CO/1% O_2_/24% H_2_ for the oxidation of CO in the presence of H_2_ (PROX), were introduced at a total flow rate of 100 mL/min (1 atm, GHSV ~ 15,000 h^−1^). The composite was heated at 1°C/min from 20 to 300°C and then cooled down at the same rate. The O_2_ and CO signals were monitored using an on-line Compact Gas Chromatograph (Interscience, Belgium) equipped with a thermal conductivity detector. O_2_ and CO conversions were determined on the basis of external calibration. Selectivity to CO_2_ is defined as the ratio between the number of mole of O_2_ used to convert CO (i.e., half of the number of mole of CO converted) over the total number of mole of O_2_ converted. It is calculated as follows: S_CO2_ = conv(CO)/2/conv(O_2_), where conv(CO) and conv(O_2_) are the conversions of CO and O_2_, respectively. It is noted that, in the H_2_-free CO oxidation reaction, the monitored consumption of oxygen is exactly half of the consumption of CO. Hence the reaction strictly follows the stoichiometry of the CO oxidation reaction (CO + ½ O_2_ CO_2_) for both catalysts, which makes the formation of CO via the Boudouart reaction (reduction of the CO_2_ product by the graphitic matrix) quite unlikely, even at high conversion levels. Besides, the CO conversion profiles in PROX show no evidence for the occurrence of the water gas shift reaction (WGS) or the reverse water gas shift reaction (RWGS) (Ivanova et al., 2010).

## Results and Discussion

### Incorporation of Copper

Sonicating a bulk graphite suspension in the presence of an aromatic copper precursor does result in the atomic scale mixing of copper and graphite in the dried composite. Table 1 shows that up to 7.8% of copper can be introduced into bulk graphite (entry 15) under our conditions and that the copper content is dependent on sonication conditions, the copper complex and the post-synthesis work-up.

#### Effect of Sonication Conditions

For CuPc, higher incorporation benefits from a longer contact time at reduced sonication power, rather than high sonication power for a short time. Table 1 (entries 1–3) indeed shows that the copper content of the dried composite measured by ICP steadily increases when the time of sonication is increased from 5–35 to 60 min (while the sonication power is at the same time decreased from 100–40 to 30 W). This highlights the dual role of the sonication energy on the composite formation. On one hand, sonication is indeed intended to destabilize graphene stacks by weakening π-π interactions between graphene layers, to enhance diffusion of the solvated CuPc in-between graphene layers and thus to maximize the accessible surface for CuPc-graphene stacking. On the other hand, it weakens the CuPc-graphene interaction that can possibly be engineered under these conditions. Hence it is expected to find an optimum in the time/power couple used for sonication to reach full incorporation of the complex. Further extending the sonication time to 120 min at low power (30 W, entry 4) leads to an apparent decrease in the copper content. Nevertheless, the maximum Cu content expected in an as-synthesized CuPc/graphite composite starting from Cu/graphite = 10 wt.% is 5.2 wt.%. Indeed, the whole complex (including the Pc ligand) is present in the dried composite, as will be shown in section Characteristics of the Interaction of Copper With the Graphitic Matrix. Besides, the Cu metal center accounts for only 11 wt.% of the CuPc complex, so that for Cu/graphite = 10 wt.%, Cu/(CuPc+graphite) = 5.2 wt.%. Hence, the ICP-derived Cu contents shown for entries 3 and 4 both suggest full copper deposition, in agreement with the colorless filtrates observed in these syntheses.

**Scheme 1:**
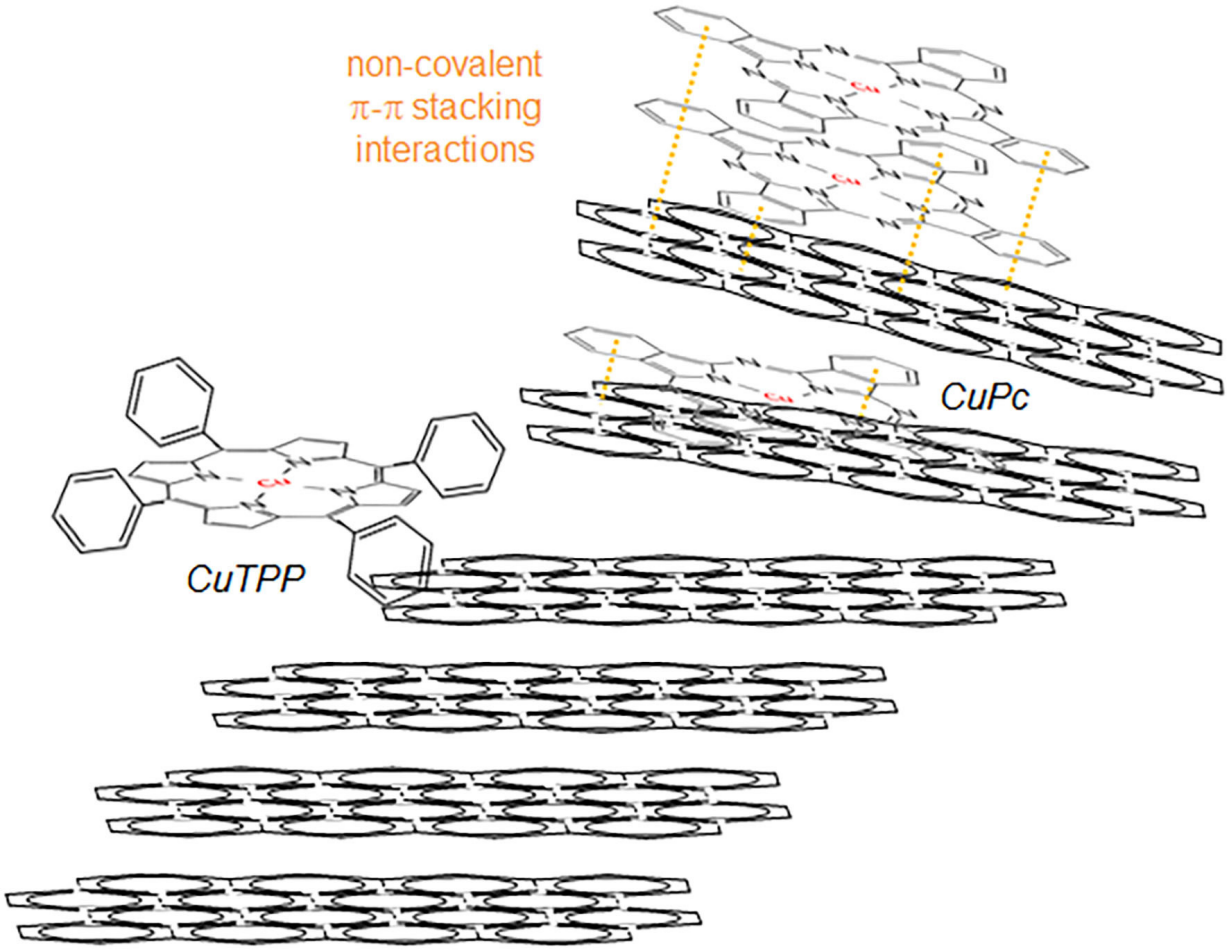
Schematic representation of the possible interactions of CuPc and CuTPP complexes with a sonically-destabilized graphitic matrix.

For CuTPP, full deposition has only been achieved with high sonication power (entry 7). Mild sonication leads to about 80% CuTPP deposition in 2 h (entry 8), considering the maximum Cu content of 4.9 wt.% expected upon full CuTPP deposition (Cu content in CuTPP is 9.4 wt.%). Further extending the sonication time (entry 9) leads to a further decrease in Cu incorporation efficiency to about 60%. Thus, in the case of CuTPP, potentially increasing diffusion of the solvated complex in-between graphene layers does not result in enhanced Cu incorporation. This is attributed to a poor porphine/graphene interaction, as compared with the CuPc/graphene interaction. Indeed, the four freely rotating phenyl groups of the CuTPP complex are expected to inhibit efficient stacking of the porphine complex to the graphene surface (Scheme 1). Sonication will thus easily break the porphine/graphene stacks, and more and more so with increasing time.

#### Effect of Copper Precursor

Comparing entries 4 and 8, it appears that the incorporation of the CuTPP complex is less efficient than that of CuPc, with only 80% CuTPP deposition observed under sonication conditions that lead to full CuPc deposition. On the other hand, comparing entries 2 and 7, it appears that deposition of the CuTPP complex under high power sonication is more efficient than that of CuPc, with full vs. 90% deposition respectively. This is attributed to differences in the geometry of the complexes. CuPc is indeed much more planar than CuTPP that contains 4 freely rotating phenyl groups regularly distributed around the porphine ring. Hence, it is expected that the 2D CuPc complex will diffuse much more efficiently within the sonically-destabilized lamellar graphitic structure than the 3D CuTPP complex which exhibits a larger cross-section. In addition, the planar CuPc complex is expected to more strongly interact with a graphene surface than CuTPP for which stacking of the porphine ring can be seriously hindered by the out-of-plane phenyl groups (Scheme 1). The introduction of CuPc thus requires milder exfoliation conditions than the introduction of CuTPP.

To preliminary understand the role of the ligands in the incorporation of copper, controlled experiments were conducted by sonicating the bulk graphite suspension in the presence of an uncomplexed copper salt, namely copper nitrate (entries 13 and 14). None of the conditions tested led to any significant incorporation of copper in the dried composite. The direct interaction between Cu^2+^ and graphene is expected to be pretty weak, as multivalent cation-π interactions are known to be much weaker than extended π-π interactions (Neel et al., 2017). It is thus consistent with the low Cu content observed in entry 13. On the other hand, conditions of entry 14 (addition of sodium borohydride to the nitrate/graphite mixture) previously allowed us to incorporate significant amounts of cobalt in graphitic matrices, using hydrolyzed NaBH_4_ derivatives as linkers (Michel et al., 2019). The much lower copper content obtained here suggests that the linkage between copper/copper oxides and graphene is not as efficient, as will be discussed in section Characteristics of the Interaction of Copper With the Graphitic Matrix.

Hence, in the case of copper, efficient incorporation in a graphitic matrix relies on the presence of complexing ligands with aromatic properties, which exhibit potential for π stacking with the aromatic graphene surface.

#### Effect of Solvent and Copper Concentration

N-methyl pyrrolidone (NMP) was previously identified as a more efficient solvent than DMF for the sonication-assisted liquid-phase exfoliation of graphite (Coleman, 2013). By comparing entries 9 and 10, it appears that replacing the DMF solvent by NMP in our synthesis actually results in a net decrease in the deposition of Cu from 60 to about 12%. This suggests that the more exfoliating NMP molecule competes more severely with CuTPP for the interaction with the graphene surface.

Decreasing the Cu/graphite ratio from 10 to 1 wt.% results in a general decrease in deposition efficiency from 100% (entries 3 and 4) down to 73 and 81% for CuPc (entries 5 and 6, respectively), and from 80 and 61% (entries 8 and 9, respectively) down to 18 and 7% for CuTPP (entries 11 and 12, respectively). Two phenomena may explain these results. First, a decrease in the concentration of the copper complex decreases the osmotic pressure within the graphitic structure, hence the diffusion of the complex within the structure and the possibility for the copper complex to compete with interlayer stacking. Second, the lower concentration also increases the competition between the copper complex and the solvent for interacting with the graphene surface, which is not in favor of the complex. Hence the particularly low Cu content of entry 12 is the result of a combination of low concentration of the copper complex, use of the highly exfoliating solvent NMP and the non-planar nature of the CuTPP complex.

### Characteristics of the Interaction of Copper With the Graphitic Matrix

#### TGA

##### Impact of complex immobilization

Oxidation of the composite in air is driven by the complex, which shifts the oxidation of graphite at much lower temperature, as shown by the following TGA study (Figure 1). TGA profiles of the composites prepared by high power sonication (entries 2 and 7) are shown in Figure 1A (plain lines). They both exhibit 2 distinct weight losses, which are attributed to oxidative degradation of the complex (lower temperature) and oxidation of the graphitic matrix (higher temperature), by comparison with TGA profiles of free complexes (dotted lines) and bare sonicated graphite (black line). In both composites, the temperature of degradation of the immobilized complex is close to that of the free complex. On the other hand, the degradation of the graphenic component occurs at much lower temperature than the oxidation of bare graphite. Temperature of half oxidation of graphite (785°C in graphite sonicated in the absence of copper compounds) is significantly decreased (Table 1), to 668°C for 5.2%CuTPP/graphite and to 647°C for 4.6%CuPc/graphite. It suggests that the oxidation of the graphitic matrix is induced by oxidative degradation of the complex, which further supports firm immobilization of the complex onto graphene. In particular, for CuPc/graphite, oxidation of graphite directly follows degradation of the complex, which clearly indicates the presence of a strong interaction between the planar Pc complex and the graphenic surface, most likely in the form of highly delocalized π interactions. The small, yet significant, temperature interval between oxidation of TPP and oxidation of graphite in CuTPP/graphite is consistent with the weaker interaction between the 2 components, due to the non-planar nature of the TPP complex. Nevertheless, both complexes induce a drastic decrease in the oxidation temperature of the graphitic matrix (>100°C). By comparison, stability of graphite (750°C) is only marginally affected by the presence of Cu in 0.02%CuNit/graphite (entry 13), likely due (1) to the absence of any strong interaction between Cu and graphite and (2) to the small Cu content.

**Figure 1:**
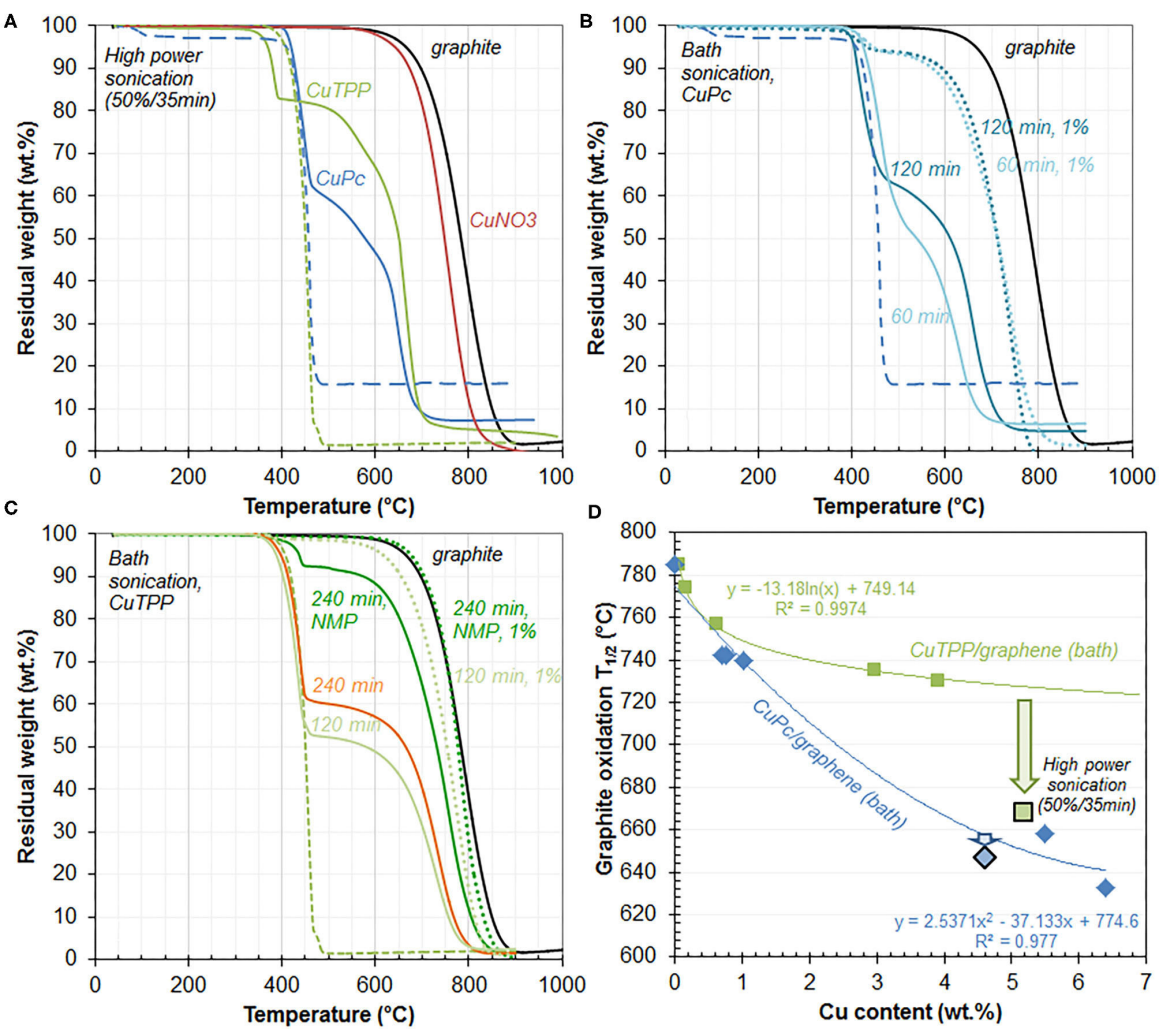
TGA profiles of high power sonication- **(A)**, CuPc- **(B)**, CuTPP- **(C)** derived composites, including sonicated graphite (50%/DMF/30 min, black line) and in **(A)**, 0.02%CuNit/graphite[20%/35 min] (plain red line), 4.6%CuPc/graphite[20%/35 min] (plain blue line), copper phthalocyanine (dotted blue line), 5.2%CuTPP/graphite[20%/35 min] (plain green line), copper tetraphenylporphine (dotted green line). In **(B)**: 6.4%CuPc/graphite[bath/60 min] (plain light blue line), 0.69%CuPc/graphite[bath/60 min/1%] (dotted light blue line), 5.5%CuPc/graphite[bath/120 min] (plain dark blue line), 0.76%CuPc/graphite[bath/120 min/1%] (dotted dark blue line). In **(C)**: 3.9%CuTPP/graphite[bath/120 min] (plain light green line), 0.16%CuTPP/graphite[bath/120 min/1%] (dotted light green line), 0.62%CuTPP/graphite [bath/240 min/NMP] (plain dark green line), 0.06%CuTPP/graphite[bath/240 min/1%/NMP] (dotted dark green line), 3.0%CuTPP/graphite[bath/240 min] (orange line). **(D)** Evolution of graphite half-oxidation temperatures as a function of Cu content for CuPc- (blue diamonds) and CuTPP- (green squares) derived composites prepared with bath sonication, as derived from Figures 1B,C, respectively. Black-lined symbols indicate data from high power sonication (Figure 1A).

##### Impact of complex loading

The impact of the complex loading on the oxidative thermal stability of the graphenic component has been studied for both CuPc/graphite (Figure 1B) and CuTPP/graphite (Figure 1C) composites obtained by mild sonication. For CuPc/graphite, high loadings (plain lines, entries 3 and 4, 6.4% and 5.5% Cu, respectively) clearly affect the oxidation of the graphenic component much more than low loadings (dotted lines, entries 5 and 6, 0.69% and 0.76% Cu, respectively). The temperature of half-degradation of graphite indeed decreases from 742°C for the low loaded composites to below 658°C for the highly loaded composites (Table 1). On the other hand, the degradation of the Pc complex, which starts at about 400°C in all cases, is unaffected by its immobilization within the composite and its loading in the composite. The decrease in graphite half-oxidation temperature with increasing CuPc loading thus results in the disappearance of the temperature interval (plateau) between the degradation of the complex and the oxidation of graphite. It is noted that the extent of the weight loss between 400 and 500°C related to degradation of the metal complex is much more pronounced for the highly loaded composites, which validates the interpretation.

Similar conclusions can be drawn for the CuTPP/graphite composites: the weight loss attributed to degradation of the CuTPP is larger for the highly loaded composites and more importantly, high CuTPP loadings (entries 8 and 9, 3.9% and 3.0% Cu, respectively) destabilize the graphenic component more than low CuTPP loadings (entries 10–12, 0.62%, 0.16%, and 0.06% Cu, respectively). However, this occurs to a lesser extent as compared with the CuPc/graphite composites: the temperature of half-oxidation of graphite and the temperature interval (plateau) between the degradation of the complex and the oxidation of graphite are both less impacted by the CuTPP content. The temperature interval actually appears quite independent from the CuTPP loading. This is overall attributed to the lower interaction between the 3D CuTPP complex and graphene as compared with the planar CuPc complex. It overall shows that the impact of the complex loading is conditioned by the strength of the complex-graphene interaction: the stronger the interaction, the stronger the impact.

This is confirmed by plotting the temperature of graphite half-oxidation vs. ICP-derived Cu content (Figure 1D). Such temperatures are systematically lower for the CuPc vs. CuTPP-containing composites. The difference is higher at higher Cu contents, due to the parabolic decrease of half oxidation temperatures vs. Cu contents for CuPc/graphite composites instead of the logarithmic decrease observed for CuTPP-containing composites. Such different variations in graphite half-oxidation temperatures as a function of the copper content are consistent with differences in the strength of interaction that the complex develops with the graphene surface. The stronger decrease in graphite half-oxidation temperatures with increasing Cu content observed for CuPc-containing composites is consistent with the stronger interaction that the 2D planar complex is able to engineer with the planar graphene surface, as compared with the 3D CuTPP complex which contains four freely rotating phenyl groups (Scheme 1).

Finally, by adding to the graph data from the composites resulting from high power sonication and derived from Figure 1A, one can see that the graphite half-oxidation temperature of 5.2%CuTPP/graphite (entry 7) is located at a much lower temperature than that expected from the logarithmic trend. It suggests that high power sonication does enhance the interaction between CuTPP and graphene, as discussed in section Effect of Copper Precursor. On the other hand, the point related to 4.6%CuPc/graphite (entry 2) is nicely integrated in the parabolic trend (Figure 1D), highlighting the similar nature of the CuPc composites whatever the sonication protocol used.

#### FTIR

**Figure 2:**
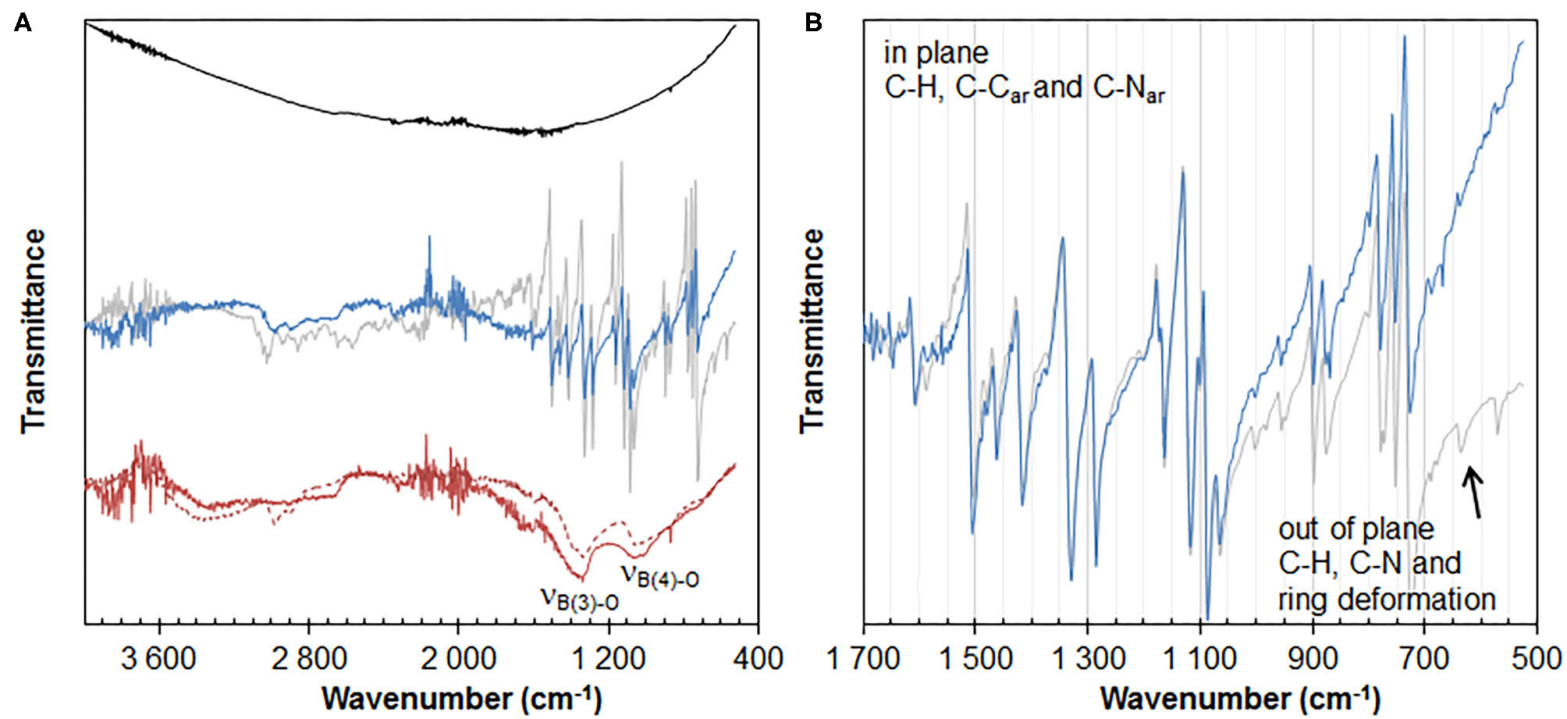
Full range **(A)** and 500–1,700 cm^−1^ range **(B)** FTIR spectra of graphite (black line), copper phthalocyanine (gray line), 3.5%CuPc/graphite[50%/5 min] (blue line), 0.7%CuNit/graphite[50%/5 min/NaBH_4_] (plain red line), and 7.5%CoNit/graphite[50%/5 min/NaBH_4_] (Michel et al., 2019) (dotted red line).

FTIR spectra of 3.5%CuPc/graphite[50%/5 min] (entry 1) and 0.7%CuNit/graphite[50%/5 min/NaBH_4_] (entry 14) are shown in Figure 2.

CuPc/graphite[50%/5 min] exhibits a well-defined vibrational pattern in the 500–1,700 cm^−1^ wavenumber region (Figure 2A) which can unambiguously be attributed to aromatic C-C, C-N, and C-H vibrations of the phthalocyanine ligand (Seoudi et al., 2005), as clearly evidenced by comparison with the FTIR spectrum of the free CuPc complex. This evidences the presence of the complex in the composite. Overall intensity of the CuPc pattern is lower in the composite than in the free CuPc. More interestingly, by normalizing the CuPc and CuPc/graphite spectra to in-plane vibrations, and more specifically to the main C-C and C-N vibrations of isoindole at 1,325 and 1,285 cm^−1^, respectively (Figure 2B), C-H out-of-plane vibrations (728, 752, 870, 896 cm^−1^) clearly appear less intense in the composite than in the free complex. Besides, ring deformation (637 cm^−1^) is lost in the FTIR spectrum of the composite. Such inhibition of out-of-plane vibrations is a clear evidence of immobilization of the complex on a surface, and more specifically of orientation of the Pc complex parallel to the graphitic surface (Basova et al., 2009). FTIR thus gives clear indication that the complex is π-stacked with graphene layers. In this configuration, C-H bonds may indeed be involved in an interaction with the graphene surface or lost by deprotonation upon interaction with the carbon surface and Pc breathing is inhibited as a result of these delocalized π interactions.

For comparison, CuNit/graphite[50%/5 min/NaBH_4_] exhibits a very similar spectrum to that of a cobalt-containing composite prepared under the same conditions. Both spectra are indeed characterized by two broad bands at 1,050 and 1,340 cm^−1^ (Figure 2A). They are attributed to asymmetric stretching vibrations of B-O bonds found in borate compounds (ν_B(4)−O_ and ν_B(3)−O_, respectively) (Jun et al., 1995). It is consistent with the presence of moieties derived from NaBH_4_ hydrolysis and oxidation in the composite, as previously discussed (Michel et al., 2019). It is likely that these species take part in metal deposition since virtually no deposition of copper (or other metal) ion is observed in the absence of these species (entry 13). Besides, the amount of borate species present in the composites seem to correlate with the metal content, since FTIR spectra normalized to the Cu and Co metal contents exhibit B-O vibrations of similar intensities. The much lower metal deposition observed for Cu (0.7 wt.%, entry 14) vs. Co (7.5 wt.%, Michel et al., 2019) under similar synthesis conditions can thus be attributed to differences in the catalytic properties of the two metals for NaBH_4_ hydrolysis and to differences in the ability of the two metals to form borate compounds from the metals themselves. In this regard, cobalt is a much more efficient catalyst for NaBH_4_ hydrolysis, which readily involves the formation of cobalt borides and borates (Demirci and Miele, 2010). On the other hand, the formation of such stable copper compounds is not observed (Glavee et al., 1994).

### Soft Oxidation of Copper

ICP, TG and FTIR analyses thus all point to efficient copper incorporation using the CuPc complex and to a strong CuPc/graphite interaction. The following sections will focus on these composites and especially on the most loaded one (entry 1). The thermo-oxidative stability of the graphitic matrix in these composites is however much lower than that of the parent graphite and an appropriate treatment had to be designed to oxidize the immobilized complex without oxidizing the graphitic matrix.

#### XRD

**Figure 3:**
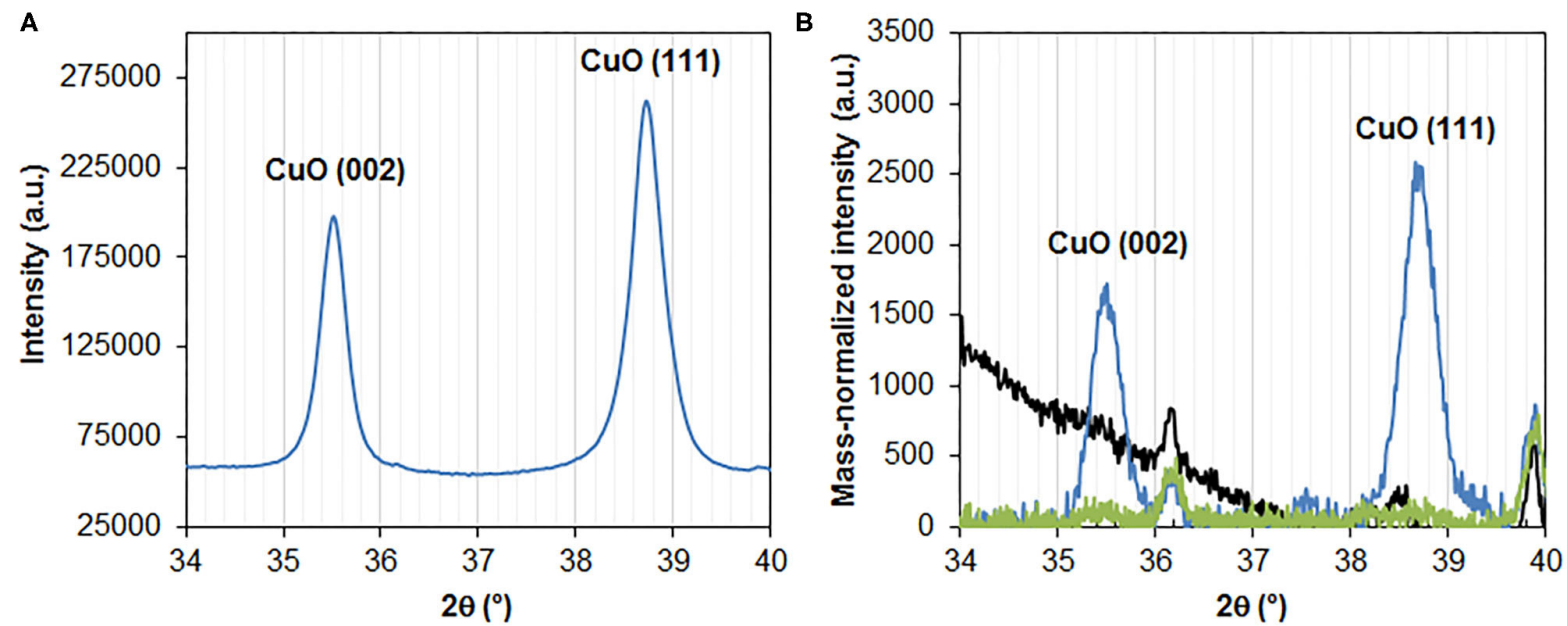
XRD of 7.8%CuPc/graphite[50%/5 min/400] **(A)** and sonicated graphite (50%/DMF/30 min, black line), 0.8%CuPc/graphite[bath/60 min/1%/450] (blue line), and 0.06%CuTPP/graphite[bath/240 min/1%/NMP/450] (green line) **(B)** in the 2θ range of 34 to 40°, showing CuO reflections.

The highly loaded CuPc composite (entry 1, 3.5% Cu) was calcined at limited temperature and time (400°C/30 min) to limit degradation of the graphitic matrix. The resulting composite (entry 15, 7.8% Cu) was analyzed by X-ray diffraction (Figure 3A). Strong reflections at 35.5 and 38.7° are observed. They correspond to the (002) and (111) reflections, respectively, of the monoclinic phase of CuO, in agreement with ICDD card No. 48-1548 and with the related literature in the field (Fuentes et al., 2010; Zhu and Diao, 2012; Du et al., 2016). The average primary crystalline domain size is calculated to be 27 ± 2 nm using the Debye-Scherrer formula. It is noted that the low loaded composite calcined at 450°C for 2 h (0.8%CuPc/graphite[bath/60 min/1%/450], entry 16) also exhibit distinct CuO-related reflections at 35.5 and 38.7° (Figure 3B). On the other hand, no Cu-related reflections is found for the calcined composite with the lowest Cu content (0.06 wt.%, entry 17) (Figure 3B).

#### TPR

**Figure 4:**
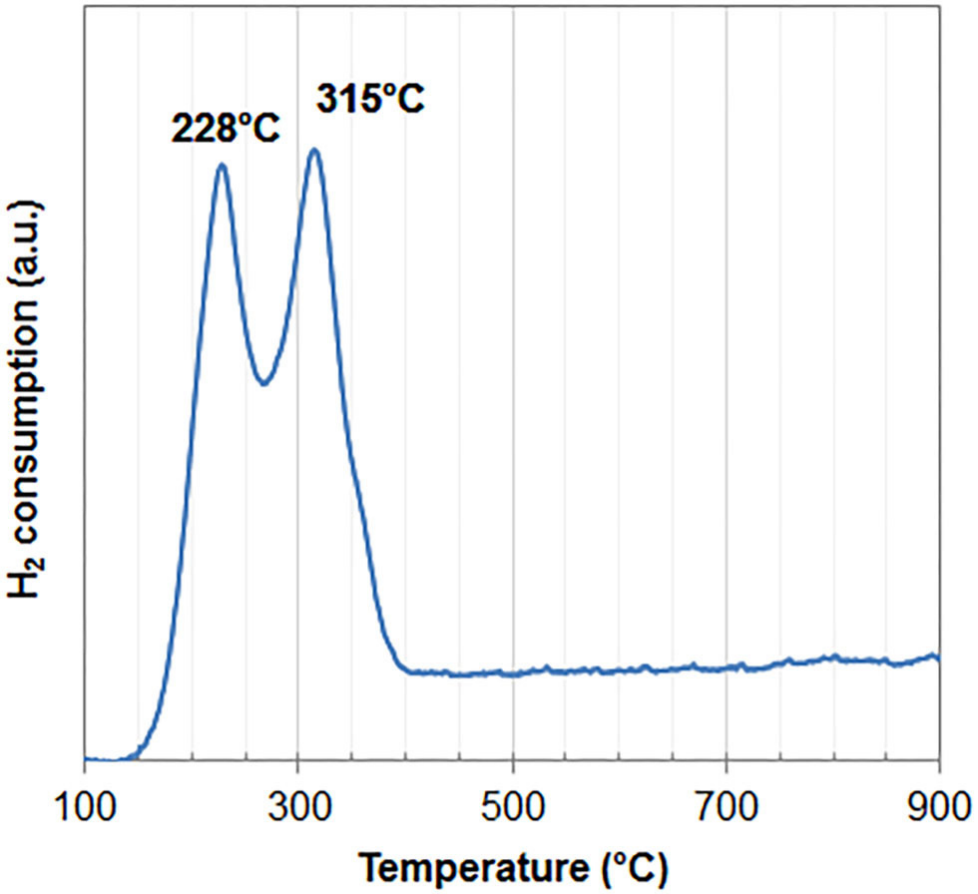
H_2_-TPR spectrum of 7.8%CuPc/graphite[50%/5 min/400].

The TPR profile of 7.8%CuPc/graphite[50%/5 min/400] (entry 15) further shows that CuO is the only copper phase present in the composite (Figure 4). The reduction profile indeed exhibits 2 main peaks at 228 and 315°C, which can be attributed to the reduction of CuO into Cu_2_O and to the reduction of Cu_2_O into Cu, respectively (Song et al., 2013; Silva et al., 2015; Ma et al., 2018). Both peaks exhibit similar intensities. Given the stoichiometry of the sequential reduction (2CuO + H_2_ → Cu_2_O + H_2_O and Cu_2_O + H_2_ → 2Cu + H_2_O), the same amount of hydrogen is needed to reduce CuO into Cu_2_O and then the produced Cu_2_O into Cu. Hence, it can be concluded that fully oxidized Cu^II^O is the sole phase of Cu that is present in the composite. The thin, well-defined peaks indicate high crystallinity of the copper oxide phase, in agreement with XRD results (section XRD) (Michel et al., 2019). Finally, the position of the low temperature reduction peak occurs at lower temperature than that usually found for bulk CuO (280°C, Silva et al., 2015). It is attributed to the reducing character of the carbonaceous support which could facilitate the reduction of the supported cupric oxide.

#### SEM

**Figure 5:**
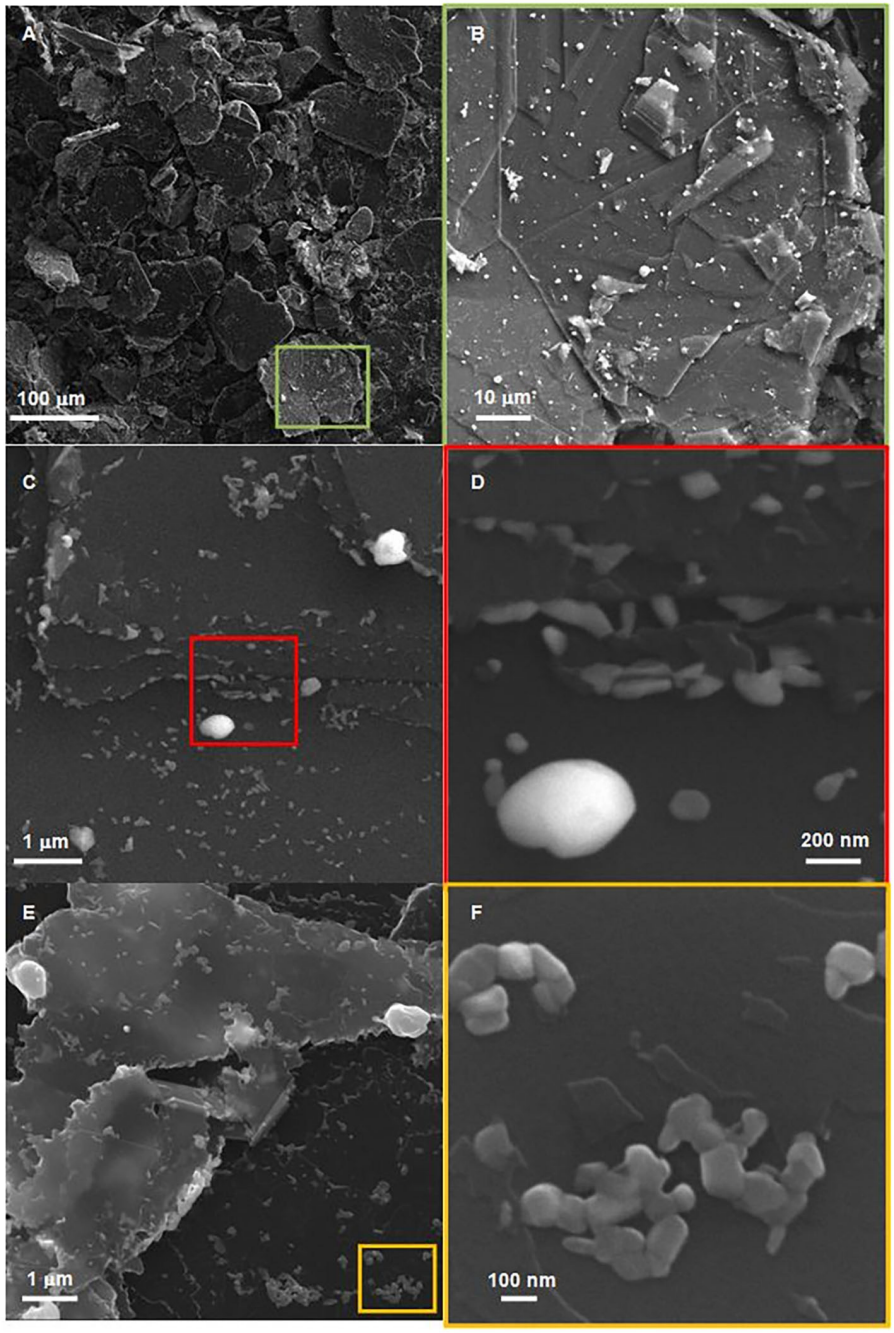
SEM images of 7.8%CuPc/graphite[50%/5 min/400] from SE **(A–D)** and InLens detectors **(E,F)**, showing CuO-decorated graphite flakes **(A)**, large CuO particles deposited on graphene **(B)**, decoration of graphene edges with smaller CuO particles **(C)**, intercalation of CuO NPs **(D)**, damaged graphene edges **(E)**, and CuO aggregates/chains **(F)**.

**Figure 6:**
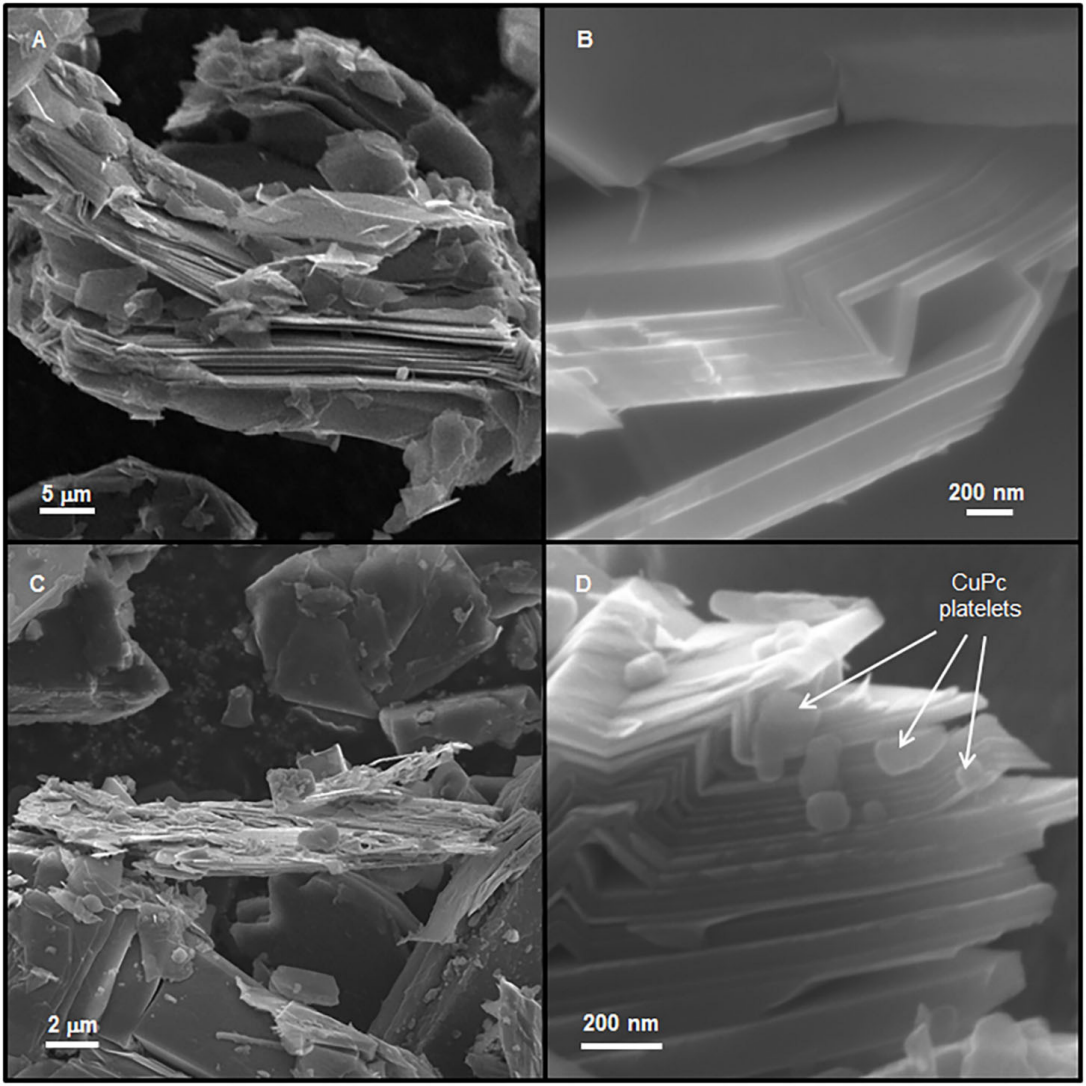
SEM images taken at 12 kV from InLens detectors of the parent graphite sonicated at 50% power in DMF for 30 min **(A,B)** (WD = 11.8 mm, AP = 60 μm) and of 3.5%CuPc/graphite[50%/5 min] **(C,D)** (WD = 11.3 mm, AP = 30 μm), showing thick stacks of graphene layers **(A)**, straight, undamaged edges **(B)**, thinner graphitic stacks **(C)**, and deposited and intercalated CuPc platelets **(D)**.

This material was further analyzed by Scanning Electron Microscopy (Figure 5). Quite high coverage of the graphitic support by CuO is observed at the micrometric scale (Figures 5A,B). CuO mostly appears in the form of aggregates/chains of 100 nm particles randomly distributed over graphite flakes (Figures 5C–F). In these areas, the Cu content determined by EDX (7.7 wt.%) is strikingly similar to the overall Cu content determined by ICP (7.8 wt.%). Huge CuO particles (500 nm−1 μm) are also present. Graphite edges appear strongly damaged in the composites (Figures 5C,E), as compared with the straight edges present in the parent graphite (Figures 6A,B). This is attributed to local oxidation of graphene layers edges upon oxidation of CuPc as indicated by the high local O content (3.5 wt.%) found by EDX, which largely exceeds that expected from CuO (7.7/63.5^*^16 = 1.94 wt.%) in those areas. It is consistent with the occurrence of CuPc adsorption close to the edges (without deep diffusion inside the graphitic matrix due to the limited reaction time of 5 min), as shown in Figures 6C,D, which is further supported by the peculiar patterns created by the spatial distribution of CuO over graphite. The in-plane dimensions of graphite flakes are similar to those observed in the copper-free parent graphite submitted to a similar sonication treatment (10–100 nm). On the other hand, thinner stacks can be found in the composites before (Figure 6C) and after oxidation, as compared with the Cu-free sonicated parent. It is attributed to insertion of copper phthalocyanine and copper oxides in-between graphite flakes (Figure 5D), which disturbs ordering of graphene layers in the direction normal to the plane.

### Copper-Induced Solid-State Exfoliation of Graphite

**Figure 7:**
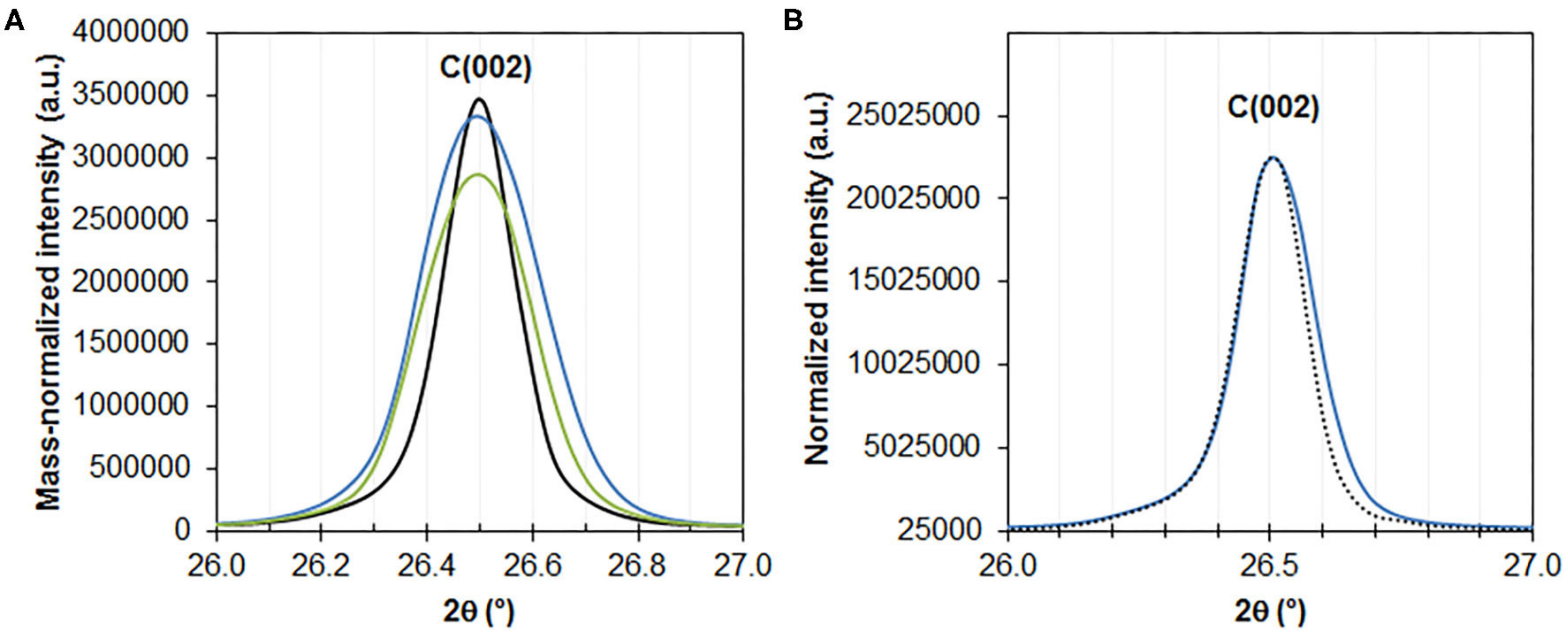
**(A)** XRD of sonicated graphite (50%/DMF/30min, black line), 0.8%CuPc/graphite[bath/60 min/1%/450] (blue line) and 0.06%CuTPP/graphite[bath/240 min/1%/NMP/450] (green line) in the 2θ range of 26 to 27°, showing the main C (002) reflection of graphite. **(B)** XRD of sonicated graphite (black line) and CuPc/graphite[50%/5 min/400] (blue line).

X-ray diffractograms of the oxidized composites all exhibit a major and dominating reflection at 26.5° which can be attributed to C(002) reflection of graphite and is due to the stacking of graphene layers in the c direction (normal to the plane). By using mass-normalized intensities, one can clearly observe broadening of the peak in the composites, as compared with bare graphite (treated under high power sonication conditions at 50% power in DMF for 30 min in the absence of any copper precursor), while the overall intensity of the peak remains similar. The full width at half-maximum appears indeed systematically larger in Cu-containing composites than in bare sonicated graphite (Figure 7A). According to the Debye-Scherrer law, this indicates that the average thickness of graphene stacks is smaller in the composites. It suggests that CuO particles and agglomerates may act as inorganic exfoliating agents, thereby decreasing the average degree of graphene stacking in the dried composite. Using the Debye-Scherrer law, this decrease can be estimated to be about 36 ± 3%. 30 nm- and 33 nm-thick stacks are indeed calculated for the 0.8%CuPc/graphite (entry 16) and 0.06%CuTPP/graphite (entry 17) oxidized composites, respectively, instead of 49 nm for bare graphite. Hence, although the exfoliation of graphite is somehow limited, as compared with the level of exfoliation achieved in the liquid phase with specifically designed techniques (Coleman, 2013), the thickness of graphite flakes may still be decreased by about one third on average in the solid state. This is in line with the low amount of CuO in these composites and the use of standard drying (instead of freeze-drying) prior to the soft oxidation. Interestingly, the highly loaded 7.8%CuO/graphite composite (entry 15) exhibits a smaller broadening of the intensity-normalized C(002) reflection (Figure 7B). It is attributed to weaker diffusion of the CuPc complex within the graphitic matrix due to limited reaction time (5 min). Hence broadening of the peak and the corresponding general mild exfoliation of graphite obtained in the solid state by mere incorporation of copper oxide nanoparticles are mainly determined by the time of sonication and in turn diffusion of the complex within the graphitic matrix, rather than bare Cu content. Still, thinner graphitic stacks may locally be observed in uncalcined 3.5%CuPc/graphite (Figure 6C) as compared with bare sonicated graphite (Figure 6A), indicating that CuPc platelets may indeed intercalate in-between graphene layers and break the stacking of the flakes.

### Catalysis

**Figure 8:**
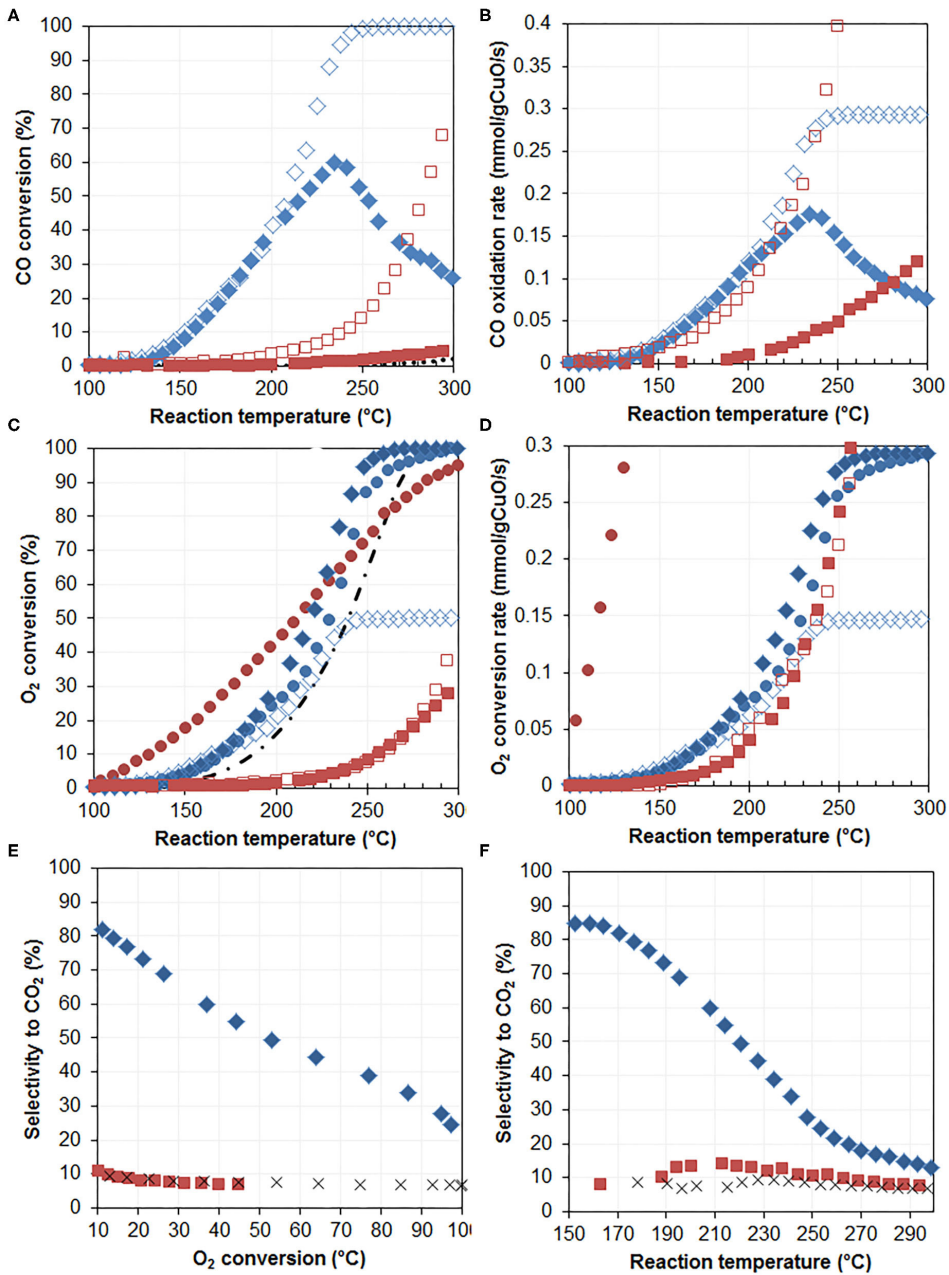
Oxidation catalysis. CO conversions **(A)**, CO oxidation rates **(B)**, oxygen conversions **(C)**, oxygen conversion rates **(D)**, selectivity to CO_2_ vs. temperature **(E)**, and vs. oxygen conversion **(F)** observed over 7.8%CuPc/graphite[50%/5 min/400] (blue diamonds) and 0.7%CuNit/graphite[50%/5 min/NaBH_4_] (red squares) in the CO+O_2_ (open symbols), CO+O_2_+H_2_ (full symbols), and H_2_+O_2_ (circles) gas-phase reactions. Black lines show the performance of bare graphite. Black line in **(C)** is for the H_2_+O_2_ reaction.

Oxidized CuPc/graphite (entry 15, 7.8% Cu) and CuNit/graphite (entry 14, 0.7% Cu) were tested for the oxidation of CO both in the absence (open symbols) and in the presence (plain symbols) of hydrogen (Figures 8A–F).

#### Performance of CuPc/graphite[50%/5 min/400]

Higher CO conversions are achieved at any given temperature, under either of the conditions, by the oxidized CuPc/graphene rather than the nitrate-derived composite (Figure 8A). This is essentially attributed to the much higher copper content of the Pc-derived catalyst, since mass-normalized CO oxidation rates of both composites (Figure 8B) under H_2_-free conditions (open symbols) are much closer. In the presence of hydrogen, oxidized CuPc/graphite exhibits similar CO conversions up to about 210°C, at which point the CO conversion decreases, due to unfavorable competition with hydrogen (CO/H_2_ = 1/24) for the limited amount of oxygen present in the feed (1%). This is generally observed in metal- (Laveille et al., 2016) and oxide- (Michel et al., 2019) catalyzed CO PROX. Nevertheless, hydrogen has virtually no impact on low temperature CO oxidation rates over oxidized CuPc/graphite. Oxygen conversions appear similar as well, up to 180°C (Figure 8C), which means that the amount of oxygen converted in the oxidation of CO under hydrogen-free conditions (CO+O_2_) is similar to the total amount of oxygen used for oxidizing both CO and H_2_ under PROX conditions (CO+O_2_+H_2_). Hence only a minor amount of hydrogen is converted below 180°C, as shown by the high selectivity to CO_2_ which remains above 80% up to 180°C (Figure 8E) and up to 15% O_2_ conversion (Figure 8F). Hydrogen starts to be significantly converted above 180°C, as indicated by both the higher oxygen conversions observed from 180°C under PROX conditions (plain diamonds) as compared with the hydrogen-free conditions (open diamonds), and the decreasing selectivity to CO_2_ with both temperature (Figure 8E) and oxygen conversion levels (Figure 8F). In the absence of CO in the feed (H_2_+O_2_ feed, blue circles), oxygen conversions are similar to those obtained in the CO+O_2_ and the CO+O_2_+H_2_ conditions up to 180°C; they remain close to those observed in CO+O_2_+H_2_ conditions above 180°C. This suggests that the overall activity of oxidized CuPc/graphite is virtually independent of the reaction conditions. This highly loaded catalyst can independently convert CO (1%) and H_2_ (24%) at the same rate, but it preferentially oxidizes CO at lower temperatures under competitive conditions (PROX). This is in marked contrast with what is observed on the low loaded CuNit/graphite catalyst.

#### Performance of CuNit/graphite [50%/5 min/NaBH_4_]

While both catalysts exhibit similar CO oxidation rates in the absence of hydrogen (Figure 8B), the nitrate-derived catalyst is much less active toward CO oxidation under PROX conditions (Figure 8B, plain squares). Nevertheless, its overall activity is not modified by the presence of hydrogen, as oxygen is converted at the same rate under CO+O_2_ (Figure 8C, open squares) and CO+O_2_+H_2_ (Figure 8C, plain squares) conditions. Besides, O_2_ conversion rates are only 2 to 3 times lower than those observed over the Pc-derived catalyst (under kinetic regime, 100–180°C temperature range in Figure 8D). The main difference is the drastically lower selectivity to CO_2_, which does not exceed 10% on the low loaded catalyst regardless of temperature and conversion levels (Figures 8E,F). This can be correlated to the orders of magnitude higher O_2_ conversion rates observed under CO-free conditions over the low loaded catalyst (Figure 8D, plain red circles), which indicate that the low loaded composite is a very efficient hydrogen oxidation catalyst. Although its overall activity is significantly inhibited by the presence of CO, it retains its strong hydrogen oxidation character and thus preferentially oxidizes H_2_ under competitive conditions (PROX). Under these conditions, the catalytic properties of the nitrate-derived catalyst actually resemble those of bare graphite, in terms of both CO conversion (Figure 8A) and selectivity (Figure 8D) levels. The oxidizing properties of copper thus appear mute/silent, turned off by the presence of hydrogen. We speculate that this may be due to the particularly low amount of copper present on the catalyst (0.7 wt.%) with regard to the reducing properties of graphite and to the reactivity of the graphene surface toward H_2_/O_2_ mixtures.

The catalytic properties of the Pc-derived composite seem on the other hand essentially controlled by the CuO phase. Intrinsic activities for CO oxidation (0.12 mmol_CO_/g_CuO_/s at 200°C) are indeed similar to those previously reported over CuO/γ-Al_2_O_3_ under optimized pre-treatment conditions (0.091 mmol_CO_/g_CuO_/s) (Wan et al., 2008). The intrinsic activity of oxidized CuPc/graphite is however significantly higher than that of air calcined CuO/γ-Al_2_O_3_ (0.02 mmol_CO_/g_CuO_/s), highlighting the relevance of the sonication-induced decoration of graphite with copper phthalocyanine in the preparation of copper oxide oxidation catalysts.

## Conclusions

The copper phthalocyanine metal complex was successfully deposited on unfunctionalized, hydrophobic, bulk graphite and used as precursor to a catalytically active CuO phase for CO oxidation, with no need for extensive exfoliation of graphite. Introduction of CuPc within the graphitic matrix is achieved by a liquid phase sonication procedure, using the DMF solvent as soft, temporary co-exfoliating agent. Immobilization of the complex onto graphene layers is evidenced in the solid state by loss of IR-active out-of-plane vibration modes of the Pc ligand. The strong hydrophobic π-π-based interaction leads to destabilization of the graphitic matrix under oxidative conditions, as shown by TGA. Destabilization of the graphitic matrix is directly related to both the strength of the interaction, as shown by comparison with the non-planar CuTPP complex, and the CuPc/graphite ratio. Controlled thermal oxidation of dried CuPc@graphite composites results in mild exfoliation of the graphitic structure. A limited, yet significant degree of exfoliation can indeed be obtained in the solid state at low Cu content (<1 wt.%), despite the low sonication power used, with composites resulting from extended sonication times. This brings indirect evidence that exfoliation is initially controlled by the diffusion of the complex within the graphitic matrix. Finally, a relatively highly loaded 9.7 wt.%CuO/graphite catalyst for CO oxidation and PROX may be obtained. It is more active than standard CuO/γ-Al_2_O_3_ catalysts, based on CuO mass-normalized reaction rates, highlighting the potential of this new method for the synthesis of oxidation catalysts.

## Data Availability Statement

The raw data supporting the conclusions of this article can be made available by the authors upon request.

## Author Contributions

GC: 9 syntheses using bath sonication, 2 calcinations, and 11 TGA. GG: 3 syntheses using tip sonication, 3 TGA, and 2 XRD. CR: 2 syntheses using tip sonication, 1 TGA and 4 FTIR. LM: 1 calcination (entry 15), 2 XRD and catalytic evaluation. VC: concept, guidance, project management, and writing of the manuscript. All authors contributed to the article and approved the submitted version.

## Conflict of Interest

The authors declare that the research was conducted in the absence of any commercial or financial relationships that could be construed as a potential conflict of interest.
